# Taxonomy of reproductive Nereididae (Annelida) in multispecies swarms at Ambon Island, Indonesia

**DOI:** 10.3897/zookeys.520.9581

**Published:** 2015-09-08

**Authors:** Joko Pamungkas, Christopher J. Glasby

**Affiliations:** 1 Research Center for Deep Sea, Indonesian Institute of Sciences, Jl. Y. Syaranamual, Guru-Guru, Poka, Ambon 97233, Indonesia; 2 Museum and Art Gallery of the Northern Territory, GPO Box 4646, Darwin NT 0801, Australia

**Keywords:** Systematics, heteronereid, epitoke, new species, Polychaeta, Maluku, Wallacia

## Abstract

Multispecies, or mass, spawning of different invertebrate species is well known for coral reef systems; however, incidences involving polychaetes are poorly documented. In this study we report on mass swarming, prior to spawning, of Nereididae at Ambon Island, Maluku, on three occasions: in 1866, inferred from an historical sample deposited in Naturalis, Leiden, and in March, 2009 and 2014, based on newly collected samples. The 2009 and 2014 events co-occurred with spawning of other polychaetes, known locally as wawo and including the widespread Indo-Pacific eunicid, *Palola viridis* (Gray in Stair). Ten species of reproductive Nereididae are described, including *Composetia marmorata* (Horst) new combination, formerly *Ceratonereis marmorata*; epitokous modifications are described for both sexes of each species including taxonomically important features such as body colour and number of pre-natatory chaetigers. Three distinct types of natatory region morphologies are recognized, which appear to characterise groups of genera. The ten new records brings to 13 the total number of nereidid species known to undergo mass swarming at Ambon Island; a key to the 13 species is provided. Species composition varies slightly between the three time periods: four species were common between all three periods, five species were in common between 1866 and 2014, and four species were in common between 1995 and 2009/14. Two species of *Neanthes* and one of *Nereis* are identified as potentially new and will be described in subsequent papers.

## Introduction

Synchronised swarming of polychaetes at the surface of the sea for the purpose of breeding is perhaps best known among the family Eunicidae, in particular the Indo-Pacific species *Palola viridis* Gray in Stair, 1847. However, swarming is also common among Nereididae and Syllidae and occurs also in at least 15 other families (Clark 1961). Two main strategies are employed: either the adult worm metamorphoses into a swimming form called an epitoke or heteronereid (many Nereididae, some Syllidae), or separate, independent reproductive individuals, called stolons, are formed (Syllidae, some Eunicidae such as *Palola* species); the epitokes and stolons then congregate (swarm) at the water surface to spawn, usually by rupture of the body wall. The epitokous strategy of Nereididae causes non-reversible modifications in adults that usually results in the death of the adult shortly after spawning. The timing of swarming in both strategies is regulated by both environmental as well as endogenous stimuli (Clark 1961; Watson et al. 2003).

In Ambon and surrounding islands of Maluku, mass swarming of polychaetes occurs every year from February to April. Locals refer to the worms as ‘wawo’ or, more popularly, as ‘laor’. The swarming has been known for a very long time, as occurring either in February and March (Rumphius 1705) or March and April (Radjawane 1982). Animals emerge from their coral substrate habitat and enter the water column two times each year, usually on the second to fourth night after a full moon immediately after sunset and continue for about two hours. In that two hour period, wawo are caught by the islanders in fishing nets (Ambonese: siru-siru) then cooked and eaten. The activity of catching the worms is known as ‘timba laor’ by the locals (Pamungkas 2011). The entire species composition of wawo from the present collections (2009, 2014), comprising 5 families and 25 species, has been listed by Pamungkas (2015); there are similar numbers of species of Eunicidae and Nereididae, but wawo is dominated in mass by the larger-bodied eunicids.

The only previous taxonomic studies of Ambonese wawo are those of Horst (1904 in English, 1905 in Dutch) and Martens et al. (1995). A small collection of epitokous worms from Ambon Island by Dutch biologist D.S. Hoedt in 1866, now at Naturalis (Leiden), was never published on, and the identity of these worms – all Nereididae – is reported in this study. The two investigations by Pamungkas (2009, 2011) were more concerned with, respectively, sexual behaviour and anthropological aspects of the worms rather than their taxonomic aspects. Since none of the above-mentioned taxonomic research was published in Indonesian (and because of their limited availability in Indonesian libraries), the natives of Ambon Island have been more influenced by Radjawane (1982), an Ambonese researcher who identified the eunicid *Lysidice oele* Horst, 1905 as the only wawo species in Maluku waters, and popularized the mass spawning phenomenon of wawo among locals. Reports of swarming Nereididae in other tropical regions are equally scarce: Hutchings and Howitt (1988) found 16 nereidid species in multiple swarming events at Lizard Island, northern Great Barrier Reeef, Australia, between October and January, 1985/86 – none of the species were identified beyond family, but a later review of the nereidids of this island (Glasby 2015) indicated that at least some species are in common with those at Ambon Island.

In this study we describe the taxonomy of the nereidid component of wawo collected in 2009/14 and nereidids in Hoedt’s collection (1866), in particular the epitokous modifications of each sex and species. Because all samples contained a small fraction of spent worms (i.e., lacking coelomic gametes), we have assumed that spawning occurred about the time the swarming worms were collected. The species composition of the two collections, almost 150 years apart, is compared with that reported by Martens et al. (1995).

## Material and methods

**Figure 1.**
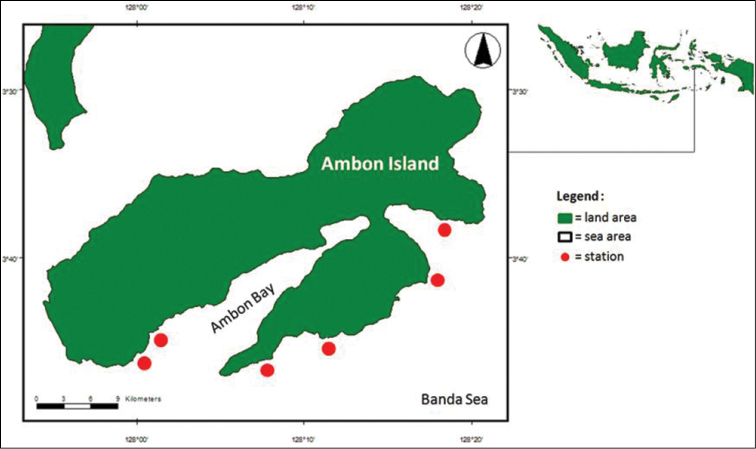
Map of Ambon Island showing location of stations, from left to right: Airlouw, Mahia, Hutumuri, Suli, Lilibooi, Alang; see Table 1 for co-ordinates and collection times.

Sexually mature polychaetes (wawo) were collected during the swarming period on 14 March, 2009 and 18–19 March, 2014, from Ambon coastal waters (Table 1, Fig. 1). At each station, the worms were collected using a small, fine-mesh hand net and were immediately fixed with 10% formaldehyde solution for at least 24 hours. A lantern or torch was used to illuminate the surroundings and attract the worms. Collected worms were rinsed with tap water to remove both the fixing agent and salt crystals, and were then preserved in 70% ethanol.

**Table 1.** Stations, collection dates and co-ordinates of *wawo* sampling.

Station	Collection dates	Coordinates
Alang	14 March 2009, 19 March 2014	3°46'18.2"S, 128°00'24.6"E
Lilibooi	19 March 2014	3°45'08.8"S, 128°01'24.6"E
Suli	18–19 March 2014	3°37'38.2"S, 128°18'25.0"E
Hutumuri	14 March 2009, 19 March 2014	3°41'27.5"S, 128°17'56.3"E
Mahia	18–19 March 2014	3°44'42.6"S, 128°11'24.6"E
Airlouw	14 March 2009, 19 March 2014	3°46'32.5"S, 128°07'53.5"E

In the laboratory, the epitokous nereidids were separated from other species of wawo by gross differences in the body form. The primary characteristics used to identify the different types of nereidid wawo were form of the parapodia and head morphology including eyes, antennae number and proboscis form. Voucher specimens were deposited at the Museum and Art Gallery of the Northern Territory, formerly Northern Territory Museum (NTM), Darwin, Australia, the Museum Zoologicum Bogoriense (MZB), Bogor, Indonesia, and the Reference Collection LIPI Ambon (RCLA), Indonesia (RCLA belongs to the Research Center for Deep Sea, Indonesian Institute of Sciences). Comparative material was sourced from the former Zoological Museum Amsterdam (ZMA) and Naturalis (formerly Rijksmuseum van Natuurlijke Historie, RMNH), Leiden; the ZMA collection is now integrated into the RMNH collection.

**Figure 2.**
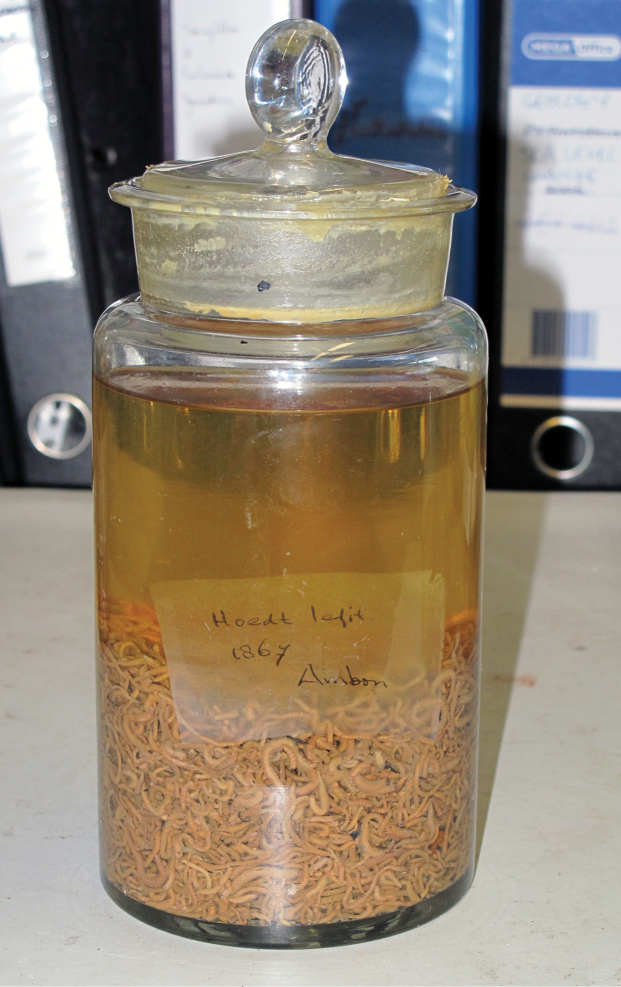
Sample of mixed-species nereidid epitokes collected Dutch biologist D.S. Hoedt in 1866, as found in Naturalis, Leiden.

Nereidid samples collected by D.S. Hoedt and V.D. Velde were studied during a visit to Naturalis (RMNH), Leiden, in 2009 by CJG. The former comprises hundreds of epitokous Nereididae in one jar (Fig. 2), which was at the time uncatalogued. The sample was received by Naturalis (formerly the Leiden Museum) in 1867 according to the register. The entire sample was studied, and selected male and female representatives of each putative species separated. A voucher collection was registered separately with the NTM.

Preserved specimens were examined using stereo (Nikon SMZ 1500 and Nikon SMZ 645) and compound (Nikon ECLIPSE 80i and Nikon ECLIPSE 50i) light microscopes. Macrophotographs of preserved animals were taken with a Canon 5D Mark II with a Canon MPE-65 Macro Lens.

Potential new species were flagged and identified using informal names or ‘cf.’; for these taxa there is currently insufficient information on morphological variation to assign a Linnean binomen. The informal species epithet takes the general format ‘colloquial name_voucher number_name of person recognising the species’, for example, *Neanthes ‘sp_Ambon_NTMW19037’* Glasby. Species are arranged alphabetically by genus and species, with informally named species at the end of each genus. Terminology for parapodial features follows Bakken and Wilson (2005) and for metamorphosed individuals (Read 2007), and for the paragnath form follows Bakken et al. (2009). Each morphospecies is diagnosed using a combination of paragnath form and pattern, epitokous modifications, and ethanol-preserved colour patterns of the body and eggs. In the absence of a robust phylogeny for Nereididae – and therefore the possibility of using a phylogenetic-based species concept – we use the morphospecies species concept as defined by Cronquist (1978), i.e., species are the smallest groups that are consistently and persistently distinct, and distinguishable by ordinary means. Our proposed species are therefore hypotheses which are falsifiable when independent data, for example morphological synapomorphies and DNA sequences, become available.

## Taxonomy

### Family Nereididae Blainville, 1818

#### *Ceratonereis* Kinberg, 1865

##### Ceratonereis singularis australis

Taxon classificationAnimaliaPhyllodocidaNereididae

Hartmann-Schröder, 1985

Fig. 3A, B

Ceratonereis (Ceratonereis) singularis australis Hartmann-Schröder, 1985: 46–47, figs 48–58.
Ceratonereis (Ceratonereis) cf. singularis australis .– Martens et al. 1995: 12, figs 3–12.
Nereis (Ceratonereis) tentaculata . – Horst 1924: 36–38, Pl. 35, figs 4–7. Non Kinberg.

###### Type locality.

Exmouth, Western Australia, Australia.

###### Material examined.

1 female (NTM W25886), 1 male (NTM W25890), 2 females (MZB.Pol.00175), 2 females (RCLA.Ann.048), all from Suli, Ambon Island, 3°37'38.2"S, 128°18'25.0"E, coll. R. Alik, 19 March 2014; 8 ex. including 5 females (NTM W23808), Banda, Maluku Province, Indonesia, coll. V.D. Velde, May 1921 (donated to NTM, formerly ZMA VPol 0962).

###### Comparative material.

1 ex.(NTM W22557), 1 ex.(NTM W23939), 1 ex.(NTM W23950), 1 ex.(NTM W23983), Lizard Island, northern Great Barrier Reef, Australia, coll. CREEFS surveys 2008–2010.

###### Size range.

Female: length (11–15 mm), maximum width (2.9–3.0 mm). Male: length (25 mm), maximum width (3.1 mm).

###### Diagnosis.

**Figure 3.**
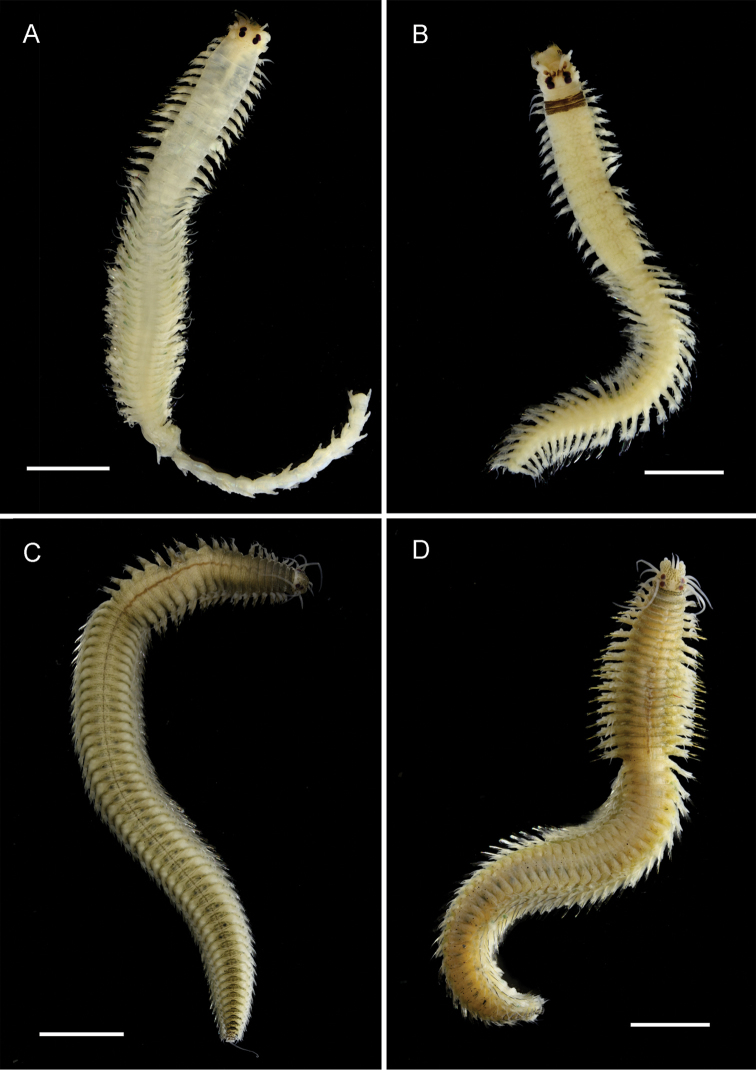
Nereidid epitokes, preserved specimens, dorsal view. **A** *Ceratonereis singularis australis*, male **B** *Ceratonereis singularis australis*, female (unmodified tail section missing) **C** *Composetia marmorata*, male **D** *Composetia marmorata*, female. Scales bars: 1 mm.

*Ceratonereis* species with females having dark brown bands on chaetiger 2 and 3 dorsally, and sometimes weaker brown bands on chaetigers 5–14; pigment apparently absent in males (1 specimen examined) (Figs 3A, B). Eyes black in Ambon specimens but purple (perhaps faded?) in Banda specimens. Paragnaths conical, arranged as follows: Areas I: 0; II: 11–16; III: 7–11; IV: 15–18; absent areas V-VIII. Apart from pigmentation, male and female epitokes identical, i.e., males lacking scalloped dorsal cirri. Pre-natatory region comprising 16–17 chaetigers; dorsal cirri of anterior chaetigers unmodified; ventral cirri of anterior chaetigers unmodified; natatory region confined to mid-body, with posterior body unmodified; pygidium not observed. Fertilised eggs adhered to body surface in some specimens, green coloured.

###### Remarks.

The specimens examined agree well with Hartmann-Schröder’s (1985) type description especially the characteristic pigmentation pattern that we observed in female specimens, and paragnath count and form of the unmodified parapodia. It is the same as the material identified by Horst (1924) as Nereis (Ceratonereis) tentaculata from eastern Indonesia and as Ceratonereis (Ceratonereis) cf. singularis australis from Ambon Island by Martens et al. (1995). Specimens of *Ceratonereis australis* from Lizard Island, northern Great Barrier Reef described by Glasby (2015) are also likely to be conspecific.

Ceratonereis (Ceratonereis) cf. perkinsi by Martens et al. (1995) appear to correspond to specimens observed by CJG from northern Australia, near the type locality (Broome, Western Australia) in terms of parapodial form and pigmentation pattern. Specimens reported by these authors as Ceratonereis (Ceratonereis) sp., do indeed belong to the *Ceratonereis* sensu stricto as described by Hartmann-Schröder (1985), as indicated by the illustrated deeply cleft prostomium (fig. 13); however, the suggestion that they are similar to *Ceratonereis japonica* is incorrect because the latter species has a very different type and arrangement of paragnaths, more in line with a species of *Solomononereis* (see Glasby 2015).

###### Distribution.

Eastern Indonesia and Australia (widespread).

#### *Composetia* Hartmann-Schröder, 1985

##### Composetia marmorata

Taxon classificationAnimaliaPhyllodocidaNereididae

(Horst, 1924) comb. n.

Fig. 3C, D

Nereis (Ceratonereis) marmorata Horst, 1924: 177–178, pl. 34, figs 13–16.

###### Type locality.

between Gisser and Seram-Laut, Maluku, Indonesia.

###### Material examined.

3 males (RCLA.Ann.042), 1 female (RCLA.Ann.043), Suli, Ambon Island, Indonesia, 3°37'38.2"S, 128°18'25.0"E, coll. R. Alik, 18 March 2014; 3 males (RCLA.Ann.044), 2 females (RCLA.Ann.045), Suli, Ambon, Indonesia, 3°37'38.2"S, 128°18'25.0"E, coll. R. Alik, 19 March 2014; 1 male (RCLA.Ann.046), Mahia, Ambon Island, Indonesia, 3°44'42.6"S, 128°11'24.6"E, coll. A.S. Leatemia, 19 March 2014; 3 males (MZB.Pol.00161), 2 females (MZB.Pol.00162), Suli, Ambon Island, Indonesia, 3°37'38.2"S, 128°18'25.0"E, coll. R. Alik, 18 March 2014; 1 male (NTM W25889), 1 female (NTM W25888), Suli, Ambon Island, Indonesia, 3°37'38.2"S, 128°18'25.0"E, coll. R. Alik, 19 March 2014; 1 ex. (NTM W23811), Salawati, Raja Ampat, Indonesia coll. unknown, 18 August 1899 (donated to NTM, formerly ZMA VPol 0980).

###### Comparative material.

Nereis (Ceratonereis) marmorata syntypes: 17 ex. (ZMA Vpol 0869) and 5 ex.(RMNH 1352), Siboga Stn. 172, Gisser anchorage, between this island and Seram- Laut, Maluku, Indonesia, 3°53'9.2"S, 130°51'56.2"E, coll. 26 August 1899. *Composetia marmorata*: 1 ex.(NTM W22530), 1 ex.(NTM W22615), 1 ex.(NTM W22797), Lizard Island, northern Great Barrier Reef, Australia, coll. CREEFS surveys 2008–2010.

###### Size range.

Male: length (20–40 mm), maximum width (3.0–5.0 mm). Female: length (17–31 mm), maximum width (2.5–4.9 mm).

###### Diagnosis.

*Composetia* species having distinctive marmorated stripes on the prostomium and anterior body (Fig. 3D). Paragnaths conical, arranged as follows: Areas I: 1–2; II: 7–14; III: 9–13; IV: 14–16; absent areas V-VIII. Male and female epitokes similar (Fig. 3C, D), except male has pygidial rosette (lacking in female) and natatory region dorsal cirri are sub-distally swollen (not scalloped) but unmodified in female; pre-natatory region comprising 17–18 chaetigers; basally swollen dorsal cirri on chaetigers 1 to 6 or 7; basally swollen ventral cirri on chaetigers 1 to 5; natatory region extends to pygidium. Female with eggs unpigmented.

###### Remarks.

The distinctive marmorated (=veined) stripes on this species of *Composetia* facilitate identification. The markings are as described by Horst (1924: 177) for Nereis (Ceratonereis) marmorata, and paragnath counts match well, although Horst reports considerable variation. Examination of paragnath numbers on the syntype material confirmed the identification. Hartmann-Schröder’s (1985) tentative allocation of the species to *Composetia* is confirmed, and formalized here. Specimens from Lizard Island, northern Great Barrier Reef described by Glasby (2015) are likely to be conspecific.

###### Distribution.

Indonesia. Lizard Island, Australia.

#### *Neanthes* Kinberg, 1865

##### Neanthes unifasciata

Taxon classificationAnimaliaPhyllodocidaNereididae

(Willey, 1905)

Fig. 4A, B

Nereis unifasciata Willey, 1905: 271–272, pl. 4, figs 85–88.

###### Type locality.

Cheval Paar, Sri Lanka

###### Material examined.

4 males (RCLA.Ann.020), 6 females (RCLA.Ann.021), Alang, Ambon Island, Indonesia, 3°46'18.2"S, 128°00'24.6"E, coll. J. Pamungkas, 19 March 2014; 10 males (RCLA.Ann.022), 11 females (RCLA.Ann.023), Lilibooi, Ambon Island, Indonesia, 3°45'08.8"S, 128°01'24.6"E, coll. J. Pamungkas, 19 March 2014; 111 males (RCLA.Ann.024), 57 females (RCLA.Ann.025), Suli, Ambon Island, Indonesia, 3°37'38.2"S, 128°18'25.0"E, coll. R. Alik, 18 March 2014; 2 females (RCLA.Ann.026), Suli, Ambon Island, Indonesia, 3°37'38.2"S, 128°18'25.0"E, coll. R. Alik, 19 March 2014; 38 males (RCLA.Ann.027), 41 females (RCLA.Ann.028), Mahia, Ambon Island, Indonesia, 3°44'42.6"S, 128°11'24.6"E, coll. A.S. Leatemia, 18 March 2014; 6 males (RCLA.Ann.029), 5 females (RCLA.Ann.030), Mahia, Ambon Island, Indonesia, 3°44'42.6"S, 128°11'24.6"E, coll. A.S. Leatemia, 19 March 2014; 3 males (MZB.Pol.00169), Mahia, Ambon Island, Indonesia, 3°44'42.6"S, 128°11'24.6"E, coll. A.S. Leatemia, 18 March 2014; 1 male (MZB.Pol.00170), Suli, Ambon Island, Indonesia, 3°37'38.2"S, 128°18'25.0"E, coll. R. Alik, 19 March 2014; 4 males (NTM W23791), 5 females (NTM W23792), Hutumuri, Ambon Island, Indonesia, 3°42.1'S, 128°17.5'E, coll. J. Pamungkas, 14 March 2009; 2 males (NTM W23796), Alang, Ambon Island, Indonesia, 3°46'18.2"S, 128°00'24.6"E, coll. J. Pamungkas, 14 March 2009; 1 ex. (specimens extracted from a jar of many hundred worms in the collection of Naturalis, Leiden) (NTM W23806), Ambon Island, Indonesia, coll. D.S. Hoedt, 1866.

###### Size range.

Male: length (15–25 mm), maximum width (1.5–2.5 mm). Female: length (18–28 mm), maximum width (2.0–3.0 mm).

###### Diagnosis.

**Figure 4.**
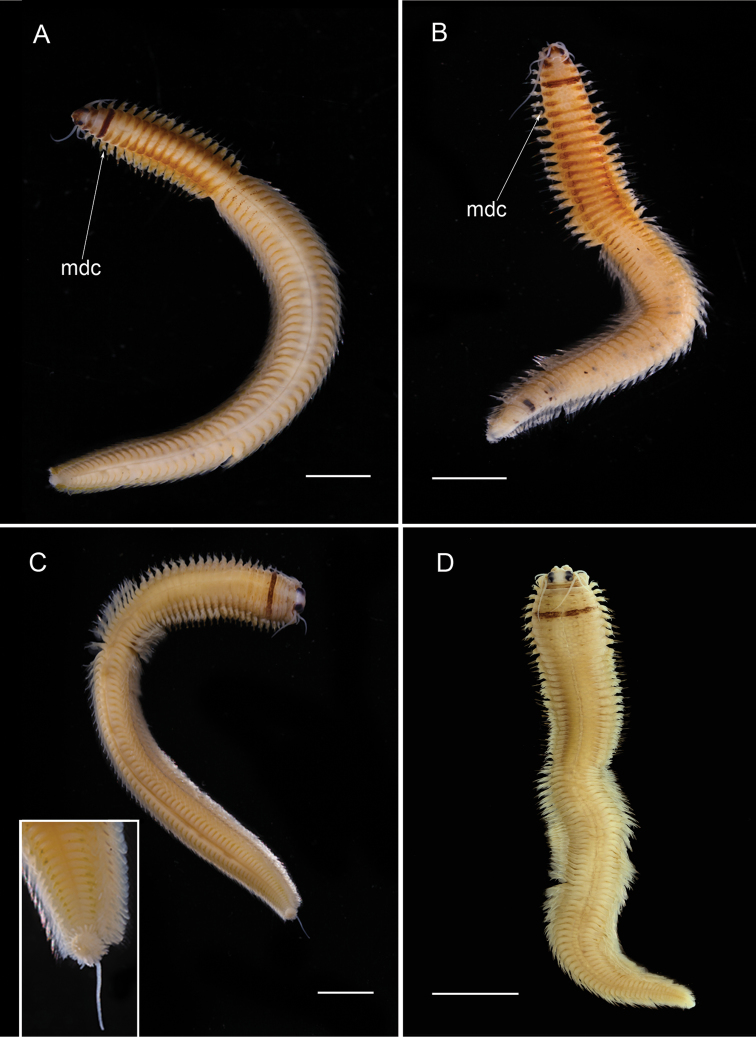
Nereidid epitokes, preserved specimens, dorsal view. **A** *Neanthes unifasciata*, male **B** *Neanthes unifasciata*, female **C** *Neanthes* sp. cf. *Neanthes gisserana* male, inset showing close up of pygidial rosette **D** *Neanthes* sp. cf. *Neanthes gisserana* female. mdc = modified dorsal cirri. Scales bars: 2 mm (**A, B**), 3 mm (**C**), 4 mm (**D**).

*Neanthes* species having dark brown band on dorsal surface of chaetiger 2 and lighter bands on following pre-natatory chaetigers (Fig. 4A, B). Paragnaths conical, arranged as follows: Areas I: 1; II: 14–16; III: 22–25; IV: 25–30; V: 0; VI: 6–12, very small: VII–VIII: 4–6 in one line. Male and female epitokes both with natatory region extending to pygidium, but differ in number of pre-natatory chaetigers and number modified dorsal and ventral cirri. Male has pre-natatory region comprising 16 chaetigers; basally swollen dorsal cirri on chaetigers 1 to 7; basally swollen ventral cirri on chaetigers 1 to 5 or 6. Female has pre-natatory region comprising 19–20 chaetigers; basally swollen dorsal cirri on chaetigers 1 to 5; basally swollen ventral cirri on chaetigers 1 to 5. Males differ from females in having subdistally swollen and scalloped dorsal cirri (smooth in females) and modified rosette pygidium.

###### Remarks.

The specimens examined in this study agree well the description of Willey (1905), which however is not very detailed. This species of *Neanthes* may be distinguished from other wawo *Neanthes* by the brown band on the dorsal side of chaetiger 2.

###### Distribution.

Indo-west Pacific (widespread).

##### Neanthes pachychaeta

Taxon classificationAnimaliaPhyllodocidaNereididae

(Fauvel, 1918)

Ceratonereis pachychaeta Fauvel, 1918: 506–508, fig. 3a–h
Neanthes pachychaeta . – Glasby, Wilson and Bakken 2011: 363–371, figs 1–7, 8d (full synonymy provided).

###### Type locality.

Djibouti and Madagascar.

###### Material examined.

Ambon Island, 1 specimen (RMNH inreg.), collected D.S. Hoedt, 1866 [specimen extracted from a jar of many hundred worms in the collection of Naturalis, Leiden; Fig. 2].

###### Size range.

Not available.

###### Remarks.

This species was recently re-described by Glasby et al. (2011); epitokes were described by Horst (1924) under the junior synonym name of Nereis (Ceratonereis) ramosa.

###### Distribution.

Indo-Pacific, widespread.

##### Neanthes sp. cf. Neanthes gisserana

Taxon classificationAnimaliaPhyllodocidaNereididae

(Horst, 1924)

Fig. 4C, D

Nereis (Lycoris) gisserana Horst, 1924: 151–152, pl. 30, figs 6, 7.

###### Material examined.

5 males (RCLA.Ann.031), 3 females (RCLA. Ann.032), Suli, Ambon Island, Indonesia, 3°37'38.2"S, 128°18'25.0"E, coll. R. Alik, 18 March 2014; 1 male (RCLA.Ann.033), 1 female (RCLA.Ann.034), Suli, Ambon Island, Indonesia, 3°37'38.2"S, 128°18'25.0"E, coll. R. Alik, 19 March 2014; 3 males (RCLA.Ann.035), 2 females (RCLA.Ann.036), Mahia, Ambon Island, Indonesia, 3°44'42.6"S, 128°11'24.6"E, coll. A.S. Leatemia, 18 March 2014; 2 males (RCLA.Ann.037), Mahia, Ambon Island, Indonesia, 3°44'42.6"S, 128°11'24.6"E, coll. A.S. Leatemia, 19 March 2014; 2 males (MZB.Pol.00167), 2 females (MZB.Pol.00168), Suli, Ambon Island, Indonesia, 3°37'38.2"S, 128°18'25.0"E, coll. R. Alik, 18 March 2014; 1 male (NTM W23797), Alang, Ambon Island, Indonesia 3°46'18.2"S, 128°00'24.6"E, coll. J. Pamungkas, 14 March 2009; 1 female (specimen extracted from a jar of many hundred worms in the collection of Naturalis, Leiden); Fig. 2 (NTM W23804), Ambon Island, Indonesia, coll. D.S. Hoedt, 1866.

###### Comparative material.

*Neanthes gisserana* (Horst, 1924). Syntypes 2 ex.(ZMA VPol 0854), Siboga Stn. 172, Gisser anchorage, between this island and Seram-Laut, Maluku, Indonesia, 18 m, coll. 26 August 1899. Neanthes cf gisserana. 1 ex.(NTM W22501), Lizard Island, northern Great Barrier Reef, Australia, coll. CREEFS surveys 2008–2010.

###### Size range.

Male: length (16–25 mm), maximum width (2.0–4.0 mm). Female: length (16–32 mm), maximum width (3.0–4.0 mm).

###### Diagnosis.

*Neanthes* species having brown band on dorsal surface of chaetiger 4 (Fig. 4C, D). Paragnaths comprise cones and bars arranged as follows: Areas I: 4 (in a longitudinal line); II: 14; III: 19–22 (including lateral groups); IV: 26 (includes cones and bars); V: 0; VI: 3–6; VII–VIII: 5–7 in transverse line. Male and female epitokes with similar body pigmentation and natatory region extending to pygidium, but differ in number of pre-natatory chaetigers and number modified dorsal and ventral cirri. Male having pre-natatory region comprising 21–22 chaetigers; basally swollen dorsal cirri on chaetigers 1 to 7; basally swollen ventral cirri on chaetigers 1 to 5 or 6. Female having pre-natatory region comprising 26 chaetigers; basally swollen dorsal cirri on chaetigers 1 to 6; basally swollen ventral cirri on chaetigers 1 to 5. Also, male has pygidial rosette (Fig. 4C) and sub-distally swollen (not scalloped) dorsal cirri, lacking in female.

###### Remarks.

The specimens examined in this study were compared with Horst's, syntypes, which they resemble closely, especially in the paragnath count and pattern; however, the present specimens are much larger than those of Horst’s, the modified chaetigers start later in the present specimens, and Horst does not mention the presence of a brown band which we observed on chaetiger 4. Therefore our material possibly represents a new species; it is very similar to specimens also referred to as *Neanthes* sp. cf *Neanthes gisserana* by Glasby (2015).

##### Neanthes sp. cf. Neanthes masalacensis

Taxon classificationAnimaliaPhyllodocidaNereididae

(Grube, 1878)

Fig. 5A, B

Nereis (Lycoris) masalacencis Grube, 1878: 75–76, pl. 5, fig. 4.

###### Material examined.

22 males (RCLA.Ann.038), 16 females (RCLA.Ann.039), Suli, Ambon Island, Indonesia, 3°37'38.2"S, 128°18'25.0"E, coll. R. Alik, 18 March 2014; 5 males (RCLA.Ann.40), 1 female (RCLA.Ann.41), Suli, Ambon Island, Indonesia, 3°37'38.2"S, 128°18'25.0"E, coll. R. Alik, 19 March 2014; 1 male (MZB.Pol.00171), 5 females (MZB.Pol.00172), Suli, Ambon Island, Indonesia, 3°37'38.2"S, 128°18'25.0"E, coll. R. Alik, 18 March 2014; 3 males (NTM W25892), 3 females (NTM W25891), Suli, Ambon Island, Indonesia, 3°37'38.2"S, 128°18'25.0"E, coll. R. Alik, 18 March 2014; Ambon Island, 4 females, 2 males (NTM W23805), collected D.S. Hoedt, 1866 (specimen extracted from a jar of many hundred worms in the collection of Naturalis, Leiden; Fig. 2).

###### Size range.

Male: length (30–47 mm), width (2.5–3.0 mm). Female: length (30–50 mm), width (2.0–3.0 mm).

###### Diagnosis.

**Figure 5.**
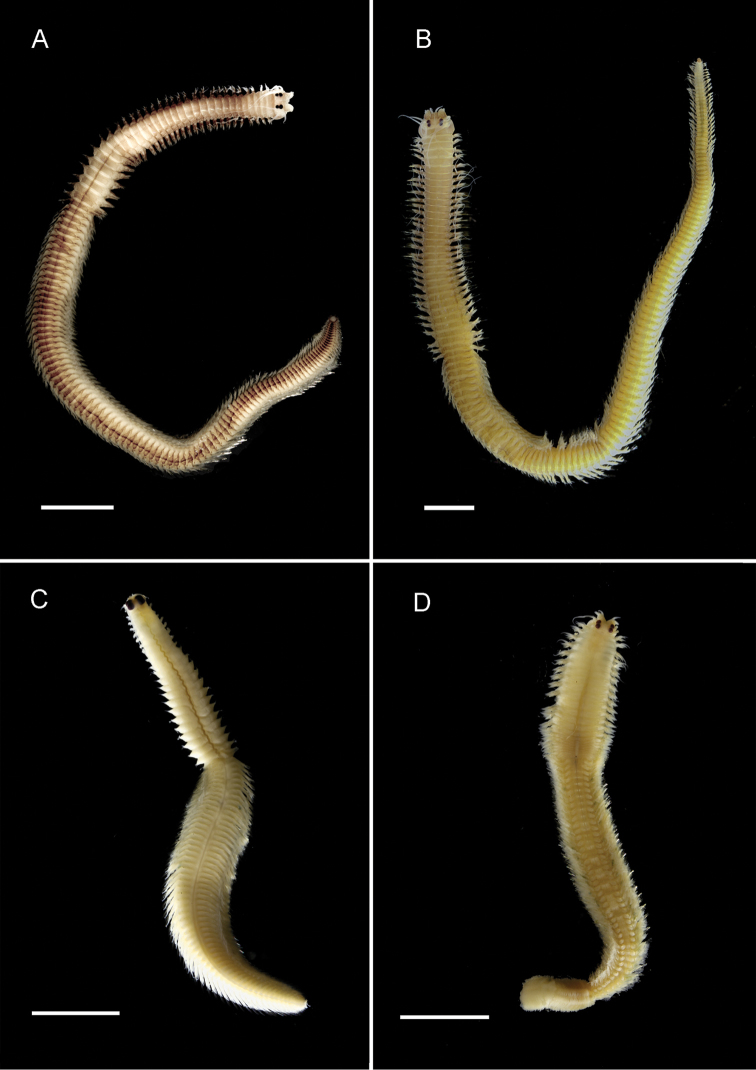
Nereidid epitokes, preserved specimens, dorsal view. **A** *Neanthes* sp. cf. *Neanthes masalacensis*, male **B** *Neanthes* sp. cf. *Neanthes masalacensis*, female **C** *Nereis ‘sp_AmbonNTMW19037*'Glasby, male **D** *Solomononereis merauensis*, female. Scales bars: 2 mm (**A, B, D**), 1 mm (**C**).

*Neanthes* species having uniform brown pigmentation on dorsal surface, apparently darker in male compared to female (Fig. 5A, B). Paragnaths conical arranged as follows: Areas I: 0; II: 10–13; III: 19–25; IV: 14–20; V: 0; VI: 1–2: VII–VII: 2–5 in line. Male and female epitokes both with natatory region extending to pygidium, but differ in number of pre-natatory chaetigers and number modified dorsal and ventral cirri. Male has pre-natatory region comprising 28–30 chaetigers; basally swollen dorsal cirri on chaetigers 1 to 7; basally swollen ventral cirri on chaetigers 1 to 6. Female has pre-natatory region comprising 32 chaetigers; basally swollen dorsal cirri on chaetigers 1 to 5; basally swollen ventral cirri on chaetigers 1 to 5. Also male has pygidial rosette and sub-distally swollen, scalloped dorsal cirri in anterior part of natatory region (dorsal cirri unmodified posteriorly), lacking in female. Female with brown pygidium.

###### Remarks.

The present material bears a general resemblance to Grube's species, which was described from Masalac, Philippines, in terms of pigmentation pattern and paragnath arrangement and counts. Our specimens differed in lacking paragnaths in Area I (Grube illustrates 2 paragnaths) and in the relatively longer dorsal cirri (up to 3 times length of parapodia in anterior chaetigers), whereas in Grube's specimens the dorsal cirri appear to be slightly longer than the parapodia, but the angle of the illustration makes it difficult to ascertain how much longer. It probably represents a new species.

Hartman (1959) referred *Neanthes masalacensis* to *Pseudonereis* based on the comparisons of earlier workers (e.g., Fauvel, Gravier) with other species of *Pseudonereis*. However, examination of the holotype of Nereis (Lycoris) masalacensis by Hutchings and Glasby (1985), showed the absence of elongated dorsal notopodial lobes in posterior chaetigers, and the original figure by Grube (1868) showed the paragnaths in Area II to be conical shaped rather than p-bars (sensu Bakken). Both features strongly suggest that the species does not belong to *Pseudonereis*. Hutchings and Glasby (1985) consider it to be indeterminable unless other material from the type locality (Masalac, Philippines) can be examined (and the distinctive pigmentation pattern matched). With this new material, from the same general biogeographic region (Western Coral Triangle) that resembles closely the type description of *Neanthes masalacensis*, we suggest the species be considered a member of *Neanthes*, as listed in Pamungkas (2015).

#### *Nereis* Linnaeus, 1858

##### Nereis ‘sp_Ambon_NTMW19037’

Taxon classificationAnimaliaPhyllodocidaNereididae

Glasby

Fig. 5C

###### Material examined.

1 female (RCLA.Ann.052), Mahia, Ambon Island, Indonesia, 3°44'42.6"S, 128°11'24.6"E, coll. A.S. Leatemia, 19 March 2014; 1 male (MZB.Pol.00173), Suli, Ambon Island, Indonesia, 3°37'38.2"S, 128°18'25.0"E, coll. R. Alik, 18 March 2014; 1 female (MZB.Pol.00174), Suli, Ambon Island, Indonesia, 3°37'38.2"S, 128°18'25.0"E, coll. R. Alik, 19 March 2014; 1 male (NTM W19155), Waimahu Beach, Ambon Island, coll. Mr Talakua, 27 March 1997; 1 male (NTM W25893), Suli, Ambon Island, Indonesia, 3°37'38.2"S, 128°18'25.0"E, coll. R. Alik, 19 March 2014.

###### Size range.

Female: length (15 mm), maximum width (2.0 mm). Male: length (16 mm), maximum width (2.0 mm).

###### Diagnosis.

*Nereis* species lacking pigmentation (Fig. 5C). Paragnaths include cones and bars arranged as follows: Areas I: 0–1 cone; II: 15–20 cones; III: 19–26 cones; IV: about 45 in large crescentic patch includes cones and bars; V: 0; VI: 15–20 small patch of minute cones; VII–VIII: one narrow band of 50–60 cones with several additional rows at right angles. Male and female epitokes both with natatory region extending to pygidium, but differ in number of pre-natatory chaetigers and number modified dorsal and ventral cirri. Male has pre-natatory region comprising 22–23 chaetigers; basally swollen dorsal cirri on chaetigers 1 to 7; basally swollen ventral cirri on chaetigers 1 to 5 or 6. Female has pre-natatory region comprising 26 chaetigers; basally swollen dorsal cirri on chaetigers 1 to 5; basally swollen ventral cirri on chaetigers 1 to 4. Also male has pygidial rosette and scalloped dorsal cirri in natatory region (except near pygidium where dorsal cirri become smooth and tapered again), rosette and scalloped dorsal cirri lacking in female.

###### Remarks.

This appears to be a new species, and will be described when further specimens, including atokous individuals become available. It was listed by Pamungkas (2015) as *Nereis* sp.

#### *Perinereis* Kinberg, 1865

##### Perinereis helleri

Taxon classificationAnimaliaPhyllodocidaNereididae

(Grube, 1878)

Fig. 6A, B

Nereis (Perinereis) helleri Grube, 1878: 81–82
Perinereis cultrifera . – Martens et al. 1995: 15. Not Grube.
Perinereis helleri . – Hutchings et al. 1991: 254–255, fig. 9a–c.

###### Type locality.

Bohol, Philippines.

###### Material examined.

4 males (RCLA.Ann.001), 2 females (RCLA.Ann.002), Alang, Ambon Island, Indonesia, 3°46'18.2"S, 128°00'24.6"E, coll. J. Pamungkas, 19 March 2014; 6 males (RCLA.Ann.003), 6 females (RCLA.Ann.004), Hutumuri, Ambon Island, Indonesia, 3°41'27.5"S, 128°17'56.3"E, coll. E. Moniharapon, 19 March 2014; 1 female (RCLA.Ann.005), Mahia, Ambon Island, Indonesia, 3°44'42.6"S, 128°11'24.6"E, coll. A.S. Leatemia, 18 March 2014; 11 males (RCLA.Ann.006), 10 females (RCLA.Ann.007), Airlouw, Ambon Island, Indonesia, 3°46'32.5"S, 128°07'53.5"E, coll. F. E. de Soysa, 19 March 2014; 5 males (MZB.Pol.00163), 5 females (MZB.Pol.00164), Airlouw, Ambon Island, Indonesia, 3°46'32.5"S, 128°07'53.5"E, coll. F. E. de Soysa, 19 March 2014; 2 males (NTM W23794), 2 females (NTM W23795), Alang, Ambon Island, Indonesia 3°46'18.2"S, 128°00'24.6"E, coll. J. Pamungkas, 14 March 2009; 2 males (NTM W23799), 1 female (NTM W23800), Airlouw, Ambon Island, Indonesia, 3°46'32.5"S, 128°07'53.5"E, coll. J. Pamungkas, 14 March 2009; 5 ex. (specimens extracted from a jar of many hundred worms in the collection of Naturalis, Leiden); Fig. 2 (RMNH unreg.), Ambon Island, coll. D.S. Hoedt, 1866.

###### Size range.

Male: length (25–47 mm), maximum width (3.0–6.0 mm). Female: length (28–70 mm), maximum width (4.0–6.0 mm).

###### Diagnosis.

**Figure 6.**
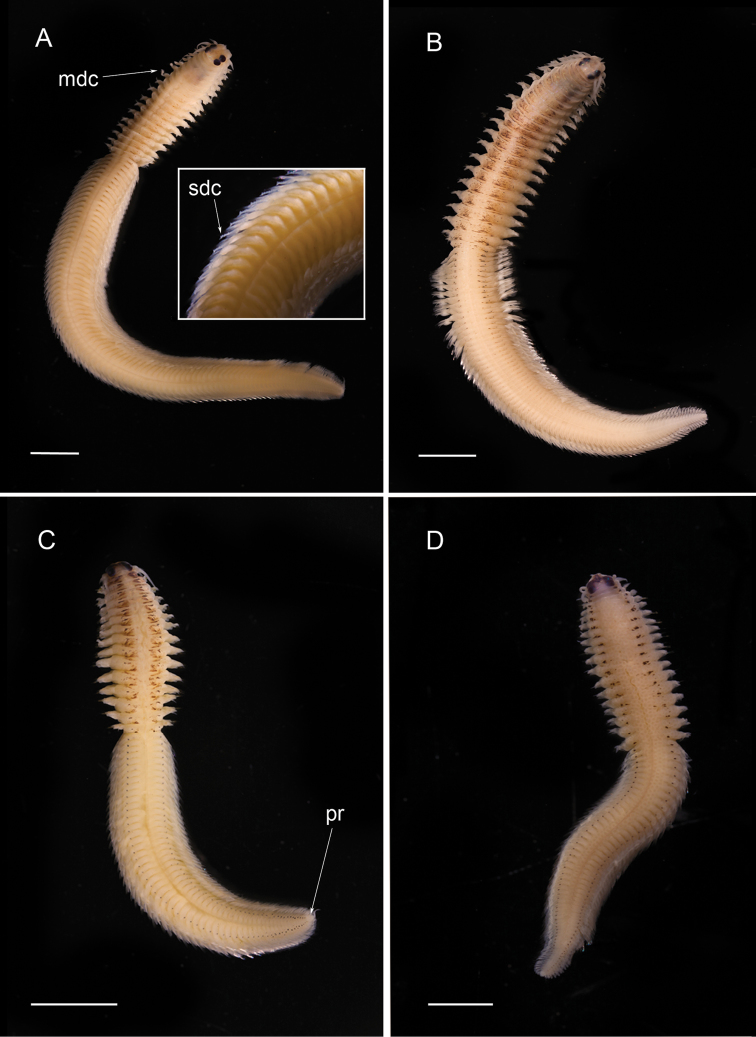
Nereidid epitokes, preserved specimens, dorsal view. **A** *Perinereis helleri*, male, inset shows close up of modified parapodia of natatory region **B** *Perinereis helleri*, female **C** *Perinereis nigropunctata*, male **D** *Perinereis nigropunctata*, female. mdc = modified dorsal cirri; sdc = scalloped dorsal cirri; pr = pygidial rosette. Scales bars: 2 mm (**A, B**), 3 mm (**C, D**).

Large *Perinereis* species having brown streaky pigmentation on dorsal surface (Fig. 6A, B). Paragnaths conical arranged as follows: Areas I: 2–3; II: 6–10; III: 14–18 (including lateral groups); IV: 17–24; V: 3; VI: 1 bar; VII–VIII: 25–30. Male and female epitokes with similar body pigmentation and natatory region extending to pygidium, but differ in number of pre-natatory chaetigers and number modified dorsal and ventral cirri. Male has pre-natatory region comprising 17 chaetigers; basally swollen dorsal cirri on chaetigers 1 to 7; basally swollen ventral cirri on chaetigers 1 to 6. Female has pre-natatory region comprising 19–20 chaetigers; basally swollen dorsal cirri on chaetigers 1 to 6; basally swollen ventral cirri on chaetigers 1 to 5. Also male has pygidial rosette, and scalloped dorsal cirri; lacking in female.

###### Remarks.

The specimens examined in this study agree well the re-description of Hutchings et al. (1991) of this species. This is one of the largest of the wawo nereidids. The specimens reported as *Perineris cultrifera* by Martens et al. (1995) are most likely *Perineris helleri*, as they fit well the paragnath number and pattern described here for this species (see also Hutchings et al. 1991), although there is a discrepancy in the number of anterior unmodified chaetigers: Martens et al. (1995) report this as 14 and 18 (male and female respectively), but our observations for this species are 17 and 19–20 (ditto). We believe Martens et al. may have confused *Perineris helleri* with *Perineris nigropunctata*, which were also found in their samples, as the latter has about 14 and 17 anterior unmodified chaetigers for male and female respectively (see following account).

###### Distribution.

Indo-Pacific (widespread).

##### Perinereis nigropunctata

Taxon classificationAnimaliaPhyllodocidaNereididae

(Horst, 1889)

Fig. 6C, D

Nereis nigropunctata Horst, 1889: 171, pl. 8, figs 1–3.
Perinereis cultrifera . – Martens et al. 1995: 15–16. Not Grube.
Perinereis nigropunctata . – Hutchings et al. 1991: 256–257, fig. 10a–e.

###### Type locality.

Malaysia.

###### Material examined.

7 males (RCLA.Ann.008), 9 females (RCLA.Ann.009), Suli, Ambon Island, Indonesia, 3°37'38.2"S, 128°18'25.0"E, coll. R. Alik, 18 March 2014; 19 males (RCLA.Ann.010), 17 females (RCLA.Ann.011), Suli, Ambon Island, Indonesia, 3°37'38.2"S, 128°18'25.0"E, coll. R. Alik, 19 March 2014; 18 males (RCLA.Ann.012), 32 females (RCLA.Ann.013), Hutumuri, Ambon Island, Indonesia, 3°41'27.5"S, 128°17'56.3"E, coll. E. Moniharapon, 19 March 2014; 1 male (RCLA.Ann.014), 4 females (RCLA.Ann.015), Mahia, Ambon Island, Indonesia, 3°44'42.6"S, 128°11'24.6"E, coll. A.S. Leatemia, 18 March 2014; 1 male (RCLA.Ann.016), 1 female (RCLA.Ann.017), Mahia, Ambon Island, Indonesia, 3°44'42.6"S, 128°11'24.6"E, coll. A.S. Leatemia, 19 March 2014; 1 male (RCLA.Ann.018), 4 females (RCLA.Ann.019), Alang, Ambon Island, Indonesia, 3°46'18.2"S, 128°00'24.6"E, coll. J. Pamungkas, 19 March 2014; 5 males (MZB.Pol.00165), 3 females (MZB.Pol.00166), Suli, Ambon Island, Indonesia, 3°37'38.2"S, 128°18'25.0"E, coll. R. Alik, 19 March 2014; 1 ex.(NTM W23793), 1 female (NTM W23798), Alang, Ambon Island, Indonesia, 3°46'18.2"S, 128°00'24.6"E, coll. J. Pamungkas, 14 March 2009; 3 females (NTM W23801), Airlouw, Ambon Island, Indonesia, 3°46'32.5"S, 128°07'53.5 E”, coll. F. E. de Soysa, 14 March 2009; 5 ex. be (specimen extracted from a jar of many hundred worms in the collection of Naturalis, Leiden) (RMNH unreg.), Ambon Island, Indonesia, coll. D.S. Hoedt, 1866.

###### Size range.

Male: length (10–20 mm), maximum width (2.0–3.0 mm). Female: length (11–23 mm), maximum width (2.0–4.0 mm).

###### Diagnosis.

*Perinereis* species having brown, streaky pigmentation on dorsal surface (Fig. 4C, D). Paragnaths conical arranged as follows: Areas I: 7–9; II: 15–21; III: 22–35 (including lateral groups); IV: 24–36; V: 3; VI: 1 bar (rarely 2 on one side); VII–VIII: 32–43. Male and female epitokes with similar body pigmentation and natatory region extending to pygidium, but differ in number of pre-natatory chaetigers and number modified dorsal and ventral cirri. Male has pre-natatory region comprising 14 chaetigers; basally swollen dorsal cirri on chaetigers 1 to 7; basally swollen ventral cirri on chaetigers 1 to 5. Female has pre-natatory region comprising 17 chaetigers; basally swollen dorsal cirri on chaetigers 1 to 5; basally swollen ventral cirri on chaetigers 1 to 5. Also male has pygidial rosette and scalloped dorsal cirri, lacking in female.

###### Remarks.

The specimens examined in this study agree well the description of Hutchings et al. (1991) of this species. This is the smaller of the two wawo *Perinereis*.

###### Distribution.

Indo-west Pacific (widespread).

#### *Solomononereis* Gibbs, 1971

##### Solomononereis merauensis

Taxon classificationAnimaliaPhyllodocidaNereididae

Gibbs, 1971

Fig. 5D

Solomononereis merauensis Gibbs, 1971: 152–153, fig. 8a–h. – Hutchings and Reid 1991: 59–60.

###### Type locality.

Solomon Islands.

###### Material examined.

1 female (MZB.Pol.00176), Suli, Ambon Island, Indonesia, 3°37'38.2"S, 128°18'25.0"E, coll. R. Alik, 19 March 2014; 1 female (NTM W25887), Suli, Ambon Island, Indonesia, 3°37'38.2"S, 128°18'25.0"E, coll. R. Alik, 19 March 2014.

###### Size range.

Male: not available. Female: length (20 mm), maximum width (3.0 mm).

###### Diagnosis.

*Solomononereis* species lacking body pigmentation (Fig. 5D). Paragnaths elongate-conical (i.e., rods), arranged 8 discrete groups as follows: Areas I: 12; II: 11; III: 3 patches with 10 in each; IV: 15–20. Female epitokes with pre-natatory region, natatory region extending to mid body, followed by highly modified tail region bearing short segments and reduced parapodia. Female has pre-natatory region comprising 23–24 chaetigers; dorsal and ventral cirri on anterior chaetigers unmodified. Female eggs are green coloured. Male epitoke unknown.

###### Remarks.

The present specimen fits the type description closely.

###### Distribution.

Indonesia, Northern Australia, Solomon Islands.

#### *Websterinereis* Pettibone, 1971

##### Websterinereis foli

Taxon classificationAnimaliaPhyllodocidaNereididae

(Fauvel, 1930)

Leptonereis foli Fauvel, 1930: 520, fig. 3.
Websterinereis foli . – Pettibone 1971: 23–25, figs 10, 11.
Nicon sp. – Martens et al. 1995: 17, figs 20–24.

###### Type locality.

Ile de Pins, New Caledonia.

###### Remarks.

The specimens reported as *Nicon* sp. by Martens et al (1995) are almost certainly *Websterinereis foli*. We reached this conclusion based on a comparison of their figures of parapodia and chaetae with those of Pettibone (1971: figs 10, 11) and their description of the pigmentation pattern compared to that described for this species by Pettibone (1971: fig. 10a) and Glasby (2015: figs 42, 43). Martens et al. (1995) are likely to have overlooked the pharyngeal (oral ring) papillae in their specimens as they are very small and only observable when the pharynx is fully everted: this explains the incorrect generic assignment.

###### Distribution.

Western Pacific, Indonesia, Lizard Island (Australia).

#### Key to reproductive Nereididae (wawo) of Ambon Island

The following key provides a means of identifying male and females of each species of Nereididae known to swarm at Ambon Island. It includes species reported both in the present study and in Martens et al. (1995).

**Table:** 

1	Body with 2 regions (largely unmodified pre-natatory region and natatory region with modified parapodia and chaetae)	**5**
–	Body with 3 regions (pre-natatory and one or two distinct natatory regions)	**2**
2	Single natatory region, restricted to mid-body	**3**
–	Two natatory regions, extending to pygidium (mid-body region with modified parapodia and oval-shaped posterior region with extremely reduced parapodia)	***Solomononereis merauensis***
3	Pre-natatory region with up to 36 chaetigers	**4**
–	Pre-natatory region with 37–40 chaetigers; unpigmented (females) or head and anterior segments red-brown lines and spots (males)	***Websterinereis foli***
4	Pre-natatory region with 14 chaetigers (female); brown pigment restricted to head region	***Ceratonereis* sp. cf. *Ceratonereis perkinsi***
–	Pre-natatory region with 16–17 chaetigers (male and female); females with dark brown bands on chaetigers 2 and 3	***Ceratonereis singularis australis***
–	Pre-natatory region with 28–33 (male) or 34–36 (female) chaetigers; brown pigment restricted to head and pygidium	***Ceratonereis* sp. sensu Martens et al. 1995**
5	Dark brown band on dorsal surface of chaetiger 2 (fainter brown bands on subsequent chaetigers); anterior region with 16 (male) and 19–20 (female) unmodified chaetigers	***Neanthes unifasciata***
–	Dark brown band on dorsal surface of chaetiger 4; anterior region with 21–22 (male) and 26 (female) unmodified chaetigers	***Neanthes* sp. cf. *Neanthes gisserana***
–	Body pigmentation not as above, or absent	**6**
6	Neuropodia of natatory region with distinctive ramified lamellae	***Neanthes pachychaeta***
–	Neuropodia of natatory region with smooth-edged lamellae	**7**
7	Female epitokes with 17 or 18 pre-natatory region chaetigers	**8**
–	Female epitokes with 19 or 20 pre-natatory region chaetigers (male 17)	***Perinereis helleri***
–	Female epitokes with about 26 pre-natatory region chaetigers (male 22–23)	***Nereis sp_Ambon_NTMW19037***
–	Female epitokes with about 32 pre-natatory region chaetigers (male 28–30)	**Neanthes cf. masalacensis**
8	Male epitokes with about 14 pre-natatory region chaetigers (female 17)	***Perinereis nigropunctata***
–	Male epitokes with 17 or 18 pre-natatory region chaetigers (female also 17–18)	***Composetia marmorata***

## Discussion

*Taxonomic utility of epitokal modifications*. Sexual dimorphism among reproductive Nereididae is well known – it includes differences in the number of basally-swollen anterior dorsal and ventral cirri, the number of pre-natatory region chaetigers, and the presence of undersurface scalloping on the natatory dorsal cirri and pygidial rosettes in males only (Schroeder and Hermans 1975 and references therein). All are useful taxonomic features at the species level, but as noted by Read (2007) they are infrequently known. The present study adds to the knowledge of these species-specific epitokal modifications for a number of different species. In addition, we found that males as a rule appear to have an equal number, or fewer, pre-natatory chaetigers compared to females. Further, possibly there exists colour sexual dimorphism in *Ceratonereis singularis australis*, as the presence of dorsal banding was absent in the only confirmed male specimen examined, but present in all females examined.

Less well known is that Nereididae appears to exhibit epitokal modification patterns at the generic level. Combining data from this study with literature information on *Platynereis* (Horst 1924) and on *Websterinereis* (Martens et al. 1995), we can recognize three types of natatory region morphologies: natatory regions extending over the mid- and posterior body to the pygidium (*Composetia*, *Neanthes*, *Nereis*, *Perinereis*, *Platynereis*; Figs 3C, D, 4A–D, 5A–C, 6A–D); natatory regions restricted to the mid-body and followed by an unmodified region extending to the pygidium (*Ceratonereis*, *Websterinereis*; Fig. 3A); and natatory regions of two types (mid-body part with typically modified parapodia; and posterior oval-shaped part with extremely reduced parapodia extending to pygidium (*Solomononereis*; Fig. 5D). Further, anterior dorsal and ventral cirri may be modified, i.e., basally swollen (*Composetia*, *Neanthes*, *Nereis*, *Perinereis*, *Platynereis*; Fig. 4A, B) or unmodified (*Ceratonereis*, *Solomononereis*). Finally, the dorsal cirri in the natatory region in males may be scalloped (some *Neanthes*, *Nereis*, *Perinereis*, *Platynereis*; Fig. 6A); or smooth (*Ceratonereis*, *Composetia*, Neanthes cf. gisserana). On the last point, our observations indicate that *Neanthes* is polymorphic for dorsal cirri scalloping; Schroeder and Hermans (1975) also noted variability in scalloping among *Nereis* species. This is further indication that the large nereidid genera such as *Neanthes* and *Nereis* are non-momophyletic, as suggested by Bakken and Wilson (2005).

Finally, some caution must be attributed to the generality of these patterns of epitokous modifications as possibly they are dependent on the state of sexual maturity of the individual and, of course, they are based on observations of very few species per genus.

*How many species?* The number of species recognised as comprising wawo in Maluku is increasing as we collect more intensively and overcome taxonomic problems. In the first publication on wawo, Rumphius (1705) considered that wawo was a single species, which he called Vermiculi Marini; however, it is clear that he recognised several different forms under this name. As this was more than a century before any formalised evolutionary thought, presumably he attributed the variation to different forms of a creature specially created. As the name Vermiculi marini was suggested about 50 years before Linnaeus's, binomial taxonomy system was in place, it is not accepted by the International Code of Zoological Nomenclature.

Rumphius (1999) describes one form that resembles a millipede:

… *about the thickness of an oaten pipe, quite like young Millipedes, of a mixed green, brown, and white, and look indeed somewhat disgusting, but these have a special name, and are not considered the true Wawo*.

And further, that the millipede form:

… *illumes at night, giving off a clear light, which makes people avoid them even more, since they share this attribute with the Millipedes*.

The millipede form may represent the shorter-bodied nereidids with their prominent parapodia; however, luminescence has, as far as we know, not been reported previously in swarming members of this family, so doubt must remain over the identity of Rumphius's, ‘millipedes’. The more abundant true wawo referred to by Rumphius (1999) as ‘like silken Floss, all entangled in small clumps’ are most likely *Palola viridis* and possibly other eunicid species (see Pamungkas 2015).

Horst (1904; 1905) also claimed that wawo is a single species, namely *Lysidice oele* (Eunicidae) based on collections from Banda waters during the Dutch Siboga Expedition (1899–1900). However, by the end of the twentieth century, Martens et al. (1995) had clarified the multispecies nature of wawo. He recognised 13 different species (five families) from Ambon Island waters with the eunicid *Palola viridis* as the dominant species; 7 out of 13 species of wawo were nereidids. Three of these species were the same as reported in this study, viz. *Ceratonereis singularis australis*, *Neanthes unifasciata* and *Perinereis nigropuncata* (Table 2). In the present study, six species of Nereididae were identified in the collections of Hoedt and nine species in the recent collections (Table 2; also Pamungkas (2015)). The Hoedt nereidids are all now in one jar (Fig. 2), so we do not know for sure whether they were collected from more than one site and/or on more than one occasion. However, assuming samples from all different periods each represented a single spawning event, the differences in species diversity between the three periods – 1866 (6 species), 1995 (7 species), 2009 and 2014 (9 species) – is entirely consistent with spatial or short term temporal variation and does probably not indicate long term change.

**Table 2.** Comparison of swarming nereidid species collected by Hoedt (1866), reported by Martens et al. (1995), and found in this study (2009/2014). See discussion for explanation of taxonomy.

Species	1866	1995	2009/2014
*Ceratonereis* sp. cf. *Ceratonereis perkinsi*	Absent	Present	Absent
*Ceratonereis singularis australis*	Present	Present	Present
*Ceratonereis sp_Martens et al.*	No	Present	Absent
*Composetia marmorata*	Absent	Absent	Present
*Neanthes* sp. cf. *Neanthes gisserana*	Present	Absent	Present
*Neanthes* sp. cf. *Neanthes masalacensis*	Absent	Absent	Present
*Neanthes pachychaeta*	Present	Absent	Absent
*Neanthes unifasciata*	Present	Present	Present
*Nereis sp_Ambon_NTMW19037*	Absent	Absent	Present
*Perinereis helleri*	Present	Present	Present
*Perinereis nigropunctata*	Present	Present	Present
*Solomononereis merauensis*	Absent	Absent	Present
*Websterinereis foli* (=*Nicon* sp. sensu Martens et al (1995))	Absent	Present	Absent

Finally, it is worth noting that wawo have been reported to swarm in islands nearby Ambon, such as Haruku and Nusalaut (JP pers. obs.) and therefore are probably widespread in Maluku Province. In the Banda Islands (south-east of Ambon Island), the natives call them ‘oele’. Further, studies of Nereididae by Horst (1889, 1924) from the ‘Malay Archipelago’ (includes present-day eastern Indonesia, Malaysia, Brunei, Timor Leste) yielded 44 nereidid species, many of which were reproductive; four species were the same as found in our recent collections (i.e., *Ceratonereis singularis australis*, *Composetia marmorata*, *Nereis gisserana* and *Perinereis nigropunctata*), with the latter two having widespread distributions throughout the western Coral Triangle (the actual number of species widespread in the region is likely to be much higher). Therefore, it is highly likely that polychaete swarming events throughout the western Coral Triangle region will be found to contain not only *Palola viridis* and other eunicids, but a range of nereidid species, including those found in this study and other species.

## Supplementary Material

XML Treatment for Ceratonereis singularis australis

XML Treatment for Composetia marmorata

XML Treatment for Neanthes unifasciata

XML Treatment for Neanthes pachychaeta

XML Treatment for Neanthes sp. cf. Neanthes gisserana

XML Treatment for Neanthes sp. cf. Neanthes masalacensis

XML Treatment for Nereis ‘sp_Ambon_NTMW19037’

XML Treatment for Perinereis helleri

XML Treatment for Perinereis nigropunctata

XML Treatment for Solomononereis merauensis

XML Treatment for Websterinereis foli
